# Value of Carbon-Ion Radiation Therapy for Breast Cancer

**DOI:** 10.1016/j.ijpt.2024.100629

**Published:** 2024-08-30

**Authors:** Bowen Yu, Kai-Wen Li, Yingyi Fan, Xiaohua Pei

**Affiliations:** 1 Galactophore Department, Beijing University of Chinese Medicine Third Affiliated Hospital, Beijing, China; 2 Department of Technology, CAS Ion Medical Technology Co., Ltd., Beijing, China; 3 Beijing University of Chinese Medicine Xiamen Hospital, Xiamen, China

**Keywords:** Particle therapy, Carbon-ion radiation therapy, Breast cancer, Nonclinical studies, Clinical studies

## Abstract

To explore the challenges and future of carbon-ion radiation therapy (CIRT) in breast cancer, we summarized the progress of nonclinical and clinical studies on CIRT for breast cancer in this review. A total of 6 nonclinical studies have been reported, which demonstrated a better effect of Carbon-ion irradiation compared with X‐ray in breast cancer cell lines (including triple-negative breast cancer and Human Epidermal Growth Factor Receptor 2-negative breast cancer). Combination with Hh inhibitor, dual tyrosine kinase inhibitor, and PARP inhibitor is promising as demonstrated in the in vitro studies. Approximately 34 patients with breast cancer went through CIRT treatment, as reported in 5 clinical studies. All studies demonstrated promising treatment effects with acceptable and manageable risks. In these studies, a total of 21 patients were reported with post-treatment response assessments, among whom 19 patients (90.48%) reported a response of complete response or partial response. The complete response rate was 66.67%. The time to complete the response ranged from 3 months to 24 months. No adverse events were observed in these studies except for grade 1 acute skin reaction in 14 out of the 21 patients (66.67%). Although the time to respond was longer than expected in some studies, the persistent responses and satisfactory safety profile provided the rationale for further research on this new therapy.

## Introduction

Breast cancer is the most commonly diagnosed cancer worldwide, with an estimated 2.3 million new cases (11.7% of all cancer sites) each year, followed by lung cancer (11.4%), colorectal cancer (10.0%), and prostate cancer (7.3%).^1^ An increasing burden of breast cancer has been observed, and over 3 million new cases and 1 million deaths every year are predicted by 2040.^2^ Although the overall 5-year survival rate is high for breast cancer, it remains the second leading cause of cancer-related deaths among women.^1^ New targeted therapies have emerged. However, resistance to therapy remains a crucial challenge for breast cancer.^3^ Besides, current treatments for some aggressive subtypes, such as triple-negative breast cancer (TNBC) and Human Epidermal Growth Factor Receptor 2 (HER2)-positive breast cancer, are limited, with poor prognosis.^5^, ^4^

Nonoperative management of breast cancer is evolving and provides disease control, with radiation therapy (RT) being the key modality. However, a radiosensitization strategy is necessary to achieve high local control rates.^6^ Given the generally favorable long-term disease control outcomes of modern breast cancer therapy, new techniques reducing the adverse effects of treatment have become the focus of recent studies. Particle radiotherapies are currently available, which show desirable outcomes and safety profiles for patients with tumors that are resistant to conventional RT and chemotherapy. Among the particle radiotherapies, carbon-ion radiation therapy (CIRT) made a better dose distribution with greater biological effects on many tumor types.^7^

CIRT utilizes carbon ions, which have a higher linear energy transfer (LET) compared to photons used in conventional RT. This higher LET results in increased ionization density along the particle track, causing more complex and irreparable DNA damage in cancer cells.^8^ Consequently, CIRT has a higher relative biological effectiveness (RBE) compared to conventional RT modalities such as photon therapy,^9^ meaning that carbon ions are more effective at killing cancer cells per unit dose than photons. This increased RBE is due to the dense ionization along the carbon ion track, causing clustered DNA damage that is more difficult for cancer cells to repair. In contrast, photon therapy causes more sparsely ionizing radiation, leading to more repairable DNA damage. The RBE of CIRT makes it particularly effective against hypoxic, radio-resistant, and densely packed tumor cells, which are often characteristic of aggressive breast cancers such as TNBC and HER2-positive subtypes.

Moreover, the physical properties of carbon ions allow a more precise dose distribution with a sharp distal fall-off, known as the Bragg peak, which spares surrounding healthy tissues and reduces collateral damage.^10^ This precision is particularly advantageous in breast cancer treatment, where critical structures such as the heart and lungs are in close proximity to the tumor site. Besides, CIRT is more effective in hypoxic (low oxygen) tumor environments, which are typically resistant to photon therapy. Carbon ions are less affected by the oxygen enhancement ratio, retaining their high RBE even in hypoxic conditions.^11^ This makes CIRT particularly advantageous for treating tumors with poor blood supply. Recent studies have indicated that CIRT may also enhance the antitumor immune response. The complex DNA damage caused by carbon ions can lead to the release of tumor antigens and the activation of immune pathways.^12^ This immunogenic cell death can potentially be exploited in combination with immunotherapy, providing a synergistic effect that enhances overall treatment efficacy.

The rationale for studying CIRT in breast cancer is grounded in these unique physical and biological properties. By providing enhanced local control and potentially reducing treatment-related toxicity, CIRT holds promise as an effective alternative therapy for patients with locally advanced or recurrent breast cancer who have limited options with conventional therapies. Furthermore, the integration of CIRT with immunotherapy and other targeted treatments could offer synergistic effects, improving overall treatment outcomes.

To explore the challenges and future of CIRT in breast cancer, we summarized the progress of nonclinical and clinical studies on CIRT for breast cancer in this review.

## Current advances

### Nonclinical studies

The search for relevant nonclinical studies was conducted using the following search terms: "carbon-ion radiotherapy," "CIRT," "breast cancer," "preclinical studies," and "animal models." Databases used for the search included PubMed, Scopus, Web of Science, and Google Scholar. The authors then reviewed the titles and abstracts to identify articles that reported nonclinical research on the anticancer effect of CIRT in breast cancer cells. A total of 6 nonclinical studies exploring the anticancer effect of CIRT were identified, and 4 of them compared CIRT with X-ray exposure (Table 1). Sai et al^13^ were the first to explore the effect of carbon-ion alone or in combination with cisplatin (CDDP) on breast cancer cell lines. Human breast cancer stem-like cells were treated with a carbon ion beam or X-ray irradiation alone or in combination with CDDP. Carbon ion beam combined with CDDP significantly suppressed colony and spheroid formation and more significantly inhibited cell cycle progression (sub-G1 arrest) compared to X-ray combined with CDDP. The results demonstrated that the carbon-ion beam combined with CDDP has a superior potential to kill TNBC stem-like cells (MDA-MB-231 and MDA-MB-453 cells) with irreparable severe DNA damage and enhanced apoptosis compared with X-ray exposure. In another study, significant upregulation of OATP1A2 expression in the hormone-dependent MCF7 cells, especially when irradiated with a low dose (0.5 Gy), was observed, indicating that carbon-ion (at a low dose of 0.5 Gy) alone or combined with OATP1A2 substrates, paclitaxel and methotrexate, can affect the expression of drug transporters in MCF-7 and MDA-MB231 cells.^14^ Konings et al^15^ demonstrated that gene expression of the Hh pathway was affected in response to carbon-ion irradiation, and combining Hh inhibition with radiation more effectively decreased MCF-7 breast cancer cell migration. Carbon ions significantly decreased PTCH1 expression at 8 hours after exposure (0.5 Gy: *P* = .0005; 2 Gy: *P* = .0024; and 4 Gy: *P* = .002). GLI3 and SUFU, both of which are Hh pathway inhibitors, exhibited a significant upregulation at 24 hours after a 2 and 4-Gy dose of carbon ions (GLI3: *P* < .0001; SUFU 2 Gy: *P* = .0003 and 4 Gy: *P* = .01). Carbon ions were also more effective than X-rays in lowering the surviving fraction, with a calculated RBE of 2.12 for carbon ions at 10% survival (RBE_10_).^15^ In 2020, it was proved that CIRT kills MDA-MB-231 and MCF-7 cells more effectively than X-ray, which might be due to the Akt/mTOR/p70S6K pathway inhibition. Carbon ion irradiation demonstrated a lower relative percentage of colonies formed than X-ray irradiation (*P* < .001 by 1-way analysis of variance). The proportion of cells in the G2/M phase was 13.23%, 28.52%, 56.25%, and 77.40% for X-ray irradiation and 14.03%, 77.44%, 79.01%, and 88.87% for carbon ion irradiation in MCF-7 cells, indicating that a more obvious increase in the G2/M phase ratio was observed in both cell lines after irradiation with carbon ions compared with X-rays (*P* < .001 by 1-way analysis of variance).^16^ In the same year, HER2-positive breast cancer stem-like cells were first tested. In this study, significantly larger-sized γH2AX foci were induced by carbon-ion beam irradiation combined with lapatinib, suggesting that a higher complexity of clustered DSB was induced by carbon-ion beam in combination with lapatinib. Caspase 3, LC3, and Beclin1 also showed significant enhancements. Together, the results showed that a combination of carbon-ion beam irradiation and dual tyrosine kinase inhibitor had a high potential to kill BT474 and SKBR3 cells via severe irreparable DNA damage, enhanced autophagy, and apoptosis.^17^ The most recent study explored a combination of carbon-ion irradiation with PARP inhibitor and demonstrated that olaparib after carbon-ion irradiation had a noticeable sensitizing effect on BCRA mut TNBC cells survival reduction.^18^ Compared with X-ray radiation, C-ion radiation required a lower dose (5 nM) of olaparib to show a significant effect in increasing the sensitivity to irradiation, suggesting that olaparib is a promising radio-sensitizer for BRCA mut TNBC in the treatment of CIRT.

**Table 1:** Nonclinical studies.

Authors	Cell lines	Interventions	Results
Sai et al^13^^a^	MDA-MB-231 and MDA-MB-453 cells	Carbon-ion alone or combined with cisplatin	Carbon-ion beam combined with cisplatin has superior potential to kill TNBC stem-like cells with irreparable severe DNA damage and enhanced apoptosis.
Zhou et al^14^	MCF7 and MDA-MB-231 cells	Carbon-ion alone or combined with OATP1A2 substrates, paclitaxel and methotrexate	Carbon-ion (at a low dose of 0.5 Gy) can affect the expression of drug transporters.
Konings et al^15^^a^	MCF-7 breast cancer cells	GLI1/2 inhibitor combined with X-ray or carbon-ion	Combining Hh inhibition with radiation more effectively decreased breast cancer cell migration compared with radiation treatment alone.
Zhang et al^16^^a^	MDA-MB-231 and MCF-7 cells	X-ray or carbon-ion	Carbon-ion kills MDA-MB-231 and MCF-7 cells more effectively than X-ray, which might result from the inhibition of the Akt/mTOR/p70S6K pathway.
Sai et al^17^^a^	BT474 and SKBR3 cell lines	Carbon-ion/X-ray alone or combined with lapatinib	The combination of carbon-ion beam irradiation and lapatinib has a high potential to kill HER2-positive breast CSCs, causing severe irreparable DNA damage, enhanced autophagy, and apoptosis.
Kawanishi et al^18^	MDA-MB-231 (BRCA wt) HCC1937 (BRCA mut) triple-negative breast cancer cells	Combining carbon-ion irradiation and olaparib (PARP inhibitor)	Carbon-ion irradiation reduced the survival rate of both cell lines in a dose-dependent manner; olaparib after carbon-ion irradiation had a noticeable sensitizing effect on HCC1937 (BRCA mut).

Abbreviations: TNBC, triple-negative breast cancer; CSC, cancer stem-like cell; HER2, Human Epidermal Growth Factor Receptor 2.

a Compared carbon-ion irradiation and X-ray exposure.

Taken together, nonclinical studies demonstrated a better effect of carbon-ion irradiation compared with X‐ray in breast cancer cell lines (including TNBC and HER2-negative breast cancer). Furthermore, a combination with Hh inhibitor, dual tyrosine kinase inhibitor, and PARP inhibitor is promising as demonstrated in the in vitro studies. Of note, Wang et al^19^ investigated the induction of nontargeted stress responses in mammary tissues by carbon or argon particles, providing evidence that carbon ions can also induce nontargeted, out-of-field induction of COX2 and DNA damages in breast tissues. These effects may pose new challenges to evaluating the risks associated with radiation exposure and understanding radiation-induced side effects.

### Clinical studies

Clinical studies investigating the efficacy and safety profile of CIRT in treating breast cancer are still limited. We searched for the following search terms: "carbon-ion radiotherapy," "CIRT," "breast cancer," "preclinical studies," and "patients." Databases used for the search included PubMed, Scopus, Web of Science, and Google Scholar. The authors then reviewed the titles and abstracts to identify articles that reported clinical practices of CIRT in breast cancer. The inclusion criteria for selecting the studies were (1) studies that specifically investigated the use of CIRT in breast cancer; (2) studies that reported clinical outcomes such as local control rates, survival rates, and treatment-related toxicity; and (3) studies published in peer-reviewed journals. After identifying relevant studies, data extraction was performed independently by 2 authors to ensure accuracy. Any discrepancies were resolved through discussion and consensus. The extracted data included patient demographics, treatment protocols, clinical outcomes, and follow-up duration.

A total of 5 clinical studies have been reported since 2014, including 2 case reports and 2 case series (Table 2). Akamatsu et al^20^ reported the world's first case of CIRT in treating breast cancer in 2014. A 50-year-old patient with breast cancer (ductal carcinoma with microinvasion foci, estrogen receptor-positive, 34bE12-positive, and PgR positive) of T1N0M0 who refused any surgery was treated with CIRT (4 fractions of 13.2 GyE) daily from Tuesday to Friday, within 1 week and followed up for 3 months. Carbon-ion radiation therapy reduced the tumor size and viability of the tumor. The pretreatment increased metabolism disappeared at 2 months after CIRT. Meanwhile, minimal adverse event (a grade 1 adverse skin reaction) was observed. In 2015, another case report showed a good example of CIRT on chemo-resistant breast cancer liver metastasis. The patient was a woman diagnosed with node-negative breast cancer. She received breast conservation therapy at the age of 46. The pathological findings indicated invasive ductal carcinoma, ER, PgR, and HER2 positive. Following a single fraction of CIRT (36-GyE), the 6-cm systemic therapy-resistant liver metastasis from breast cancer gradually decreased, and fluorodeoxyglucose abnormal accumulation disappeared at 8 months, without local recurrence for 8.5 years.^21^ To investigate the best irradiation method, Matsubara et al^22^ retrospectively analyzed the dose distribution of 11 Japanese patients with breast cancer who received CIRT. The median age of the 11 patients was 66 (range: 44-81). The median major axis of the tumor was 14 mm (range: 4-20), with a median distance to skin of 15 mm (range: 5-30 mm). It turned out that the *P* value obtained when comparing passive and scanning irradiation methods was .58 when a 2-sided Wilcoxon signed-rank test was employed for comparison, suggesting that the spot scanning irradiation method was not always superior to the passive method in breast cancer, which may due to the simplicity of the organ at risk and the shallow target point.^22^ Karasawa et al^23^ conducted the first phase I clinical trial of CIRT for breast cancer and reported primary results from 7 patients in 2019. The age of patients enrolled ranged from 61 to 81, with a median tumor size of 13 mm (range: 4-20 mm). These patients with low-risk stage I breast cancer were treated with CIRT (48.0, 52.8, or 60.0 GyE within 1 week) and underwent tumor excision for pathological evaluation 3 months after treatment. Grade 1 acute skin reaction was the only adverse effect (observed in 4 out of 7 cases). One complete response, 5 partial responses, and 1 stable disease were achieved in the 3-month tumor assessment. However, only 2 of the 7 patients reached grade 3 per Oboshi-Shimosato classification in the 3-month pathological evaluation. All patients were alive without recurrence or late adverse reactions in a follow-up period of 37 to 48 months.^23^ Besides these patients under the clinical trial, they reported 14 cases treated outside the clinical trial in 2020.^24^ The ages of these patients ranged from 44 to 79, with 2 of them having TNBC. The median tumor size was 14 mm (ranging from 9 to 18 mm). The median follow-up period was 61 months. Most of these (13 out of 14) patients achieved complete response, and the time to complete response ranged from 3 months to 24 months. Only 1 patient experienced tumor recurrence, who was 72 years old with triple-negative and high Ki-67 breast cancer. The only adverse effect in this study was acute skin reaction of grade 1, which was reported in 10 patients.

**Table 2:** Clinical studies.

Studies	Study types	Population	Interventions	Results	Follow-up
Akamatsu et al^20^	Case report	Early-stage breast cancer (*N* = 1)	CIRT (52.8 GyE^a^)	*Efficacy*: tumor size reduced, tumor variability decreased, pretreatment increased metabolism disappeared *Safety*: grade 1 adverse skin reaction	3 mo
Harada et al^21^	Case report	Resistant liver metastasis from breast cancer (*N* = 1)	CIRT (36.0 GyE with respiratory gating) single shot	*Efficacy*: tumor size decreased, FDG abnormal accumulation disappeared 8 mo after CIRT, and disease remained stable 8.5 y after CIRT *Safety*: not reported	8.5 y
Matsubara et al^22^	Case series	Early-stage breast cancer (*N* = 11)	CIRT (48.0, 52.8, or 60.0 GyE^a^)	The scanning irradiation method was not always superior to the passive method in dose distribution.	NA
Karasawa et al^23^	Phase I clinical study	Low-risk stage I breast cancer (*N* = 7)	CIRT (48.0, 52.8, or 60.0 GyE^a^) combined with endocrine therapy	*Efficacy*: 4 cases achieved grade 2b or more, and 2 cases reached grade 3 in the 3-mo pathological evaluation *Safety*: grade 1 acute skin reaction in 4 patients.	37-48 mo
Karasawa et al^24^	Case series	Untreated inoperable stage I breast cancer (*N* = 14)	CIRT (52.8 or 60.0 GyE^a^) alone or combined with endocrine therapy	*Efficacy*: Most patients (13 out of 14) achieved complete response in 3-24 mo after treatment. *Safety*: grade 1 acute skin reaction in 10 patients.	61 mo

Abbreviation: CIRT, carbon-ion radiation therapy; FDG, fluorodeoxyglucose.

a Doses were given in 4 days within 1 week.

In the above studies, approximately 34 patients with breast cancer went through CIRT treatment. All studies demonstrated promising treatment effects with acceptable and manageable risks. Even though some studies reported a longer duration to achieve a complete response than initially predicted, the consistent effectiveness of these responses and an adequate safety profile form a solid basis for the continued investigation of this novel treatment.

To further evaluate the effect and safety of CIRT for breast cancer, we summarized baseline characteristics and endpoints of patients with available response assessment (Table 3). Among the 21 patients who had post-treatment response assessment, 19 patients (90.48%) reported a response of complete response or partial response. The complete response rate of 66.67% is promising as compared with the local control rate of 45% to 57% of conventional radiation therapies.^6^ No adverse events were observed except for grade 1 acute skin reaction.

**Table 3:** Clinical outcomes of CIRT for breast cancer.

Baseline characteristics and endpoints	Patients (*N* = 21)
*Baseline characteristics*	
Age (years)	44-81
Sex (male/female)	0/21
Tumor size (mm)	4-20
Breast cancer subtypes	
Triple-negative breast cancer	2
Luminal A	7
Luminal B	5
ER-positive/HER2-negative	7
Stage at diagnosis	
Stage I	21
*Efficacy endpoints*	
Follow-up time (months)	37-87
Overall response rate, *n*/*N* (%)	19/21 (90.48%)
Complete response	14/21 (66.67%)
Partial response	5/21 (23.81%)
Stable disease	1/21 (4.76%)
Progressive disease	1/21 (4.76%)
*Safety endpoints*	
Adverse event	
Acute skin reaction grade 1	14/21 (66.67%)

Abbreviation: CIRT, carbon-ion radiation therapy; HER2, Human Epidermal Growth Factor Receptor 2; ER, estrogen receptor.

## Future and challenges of carbon-ion radiation therapy for breast cancer

Given the relatively favorable disease control rates of breast cancer, nonsurgical therapy, and methods to reduce adverse effects have gained considerable focus in recent investigations.^6^

Carbon-ion radiation therapy has a superior dose distribution compared to conventional RT, which provides both physical and biological advantages in the treatment of breast cancers. Physically, CIRT delivers a substantial dose to the tumoral area with minimal dose to the surrounding tissue. Biologically, CIRT increases DNA damage, autophagy, and apoptosis of tumor cells.^17^ Clinical radiologists have been performing CIRT to treat patients with breast cancers that are resistant to conventional photon RT. Primary clinical studies demonstrated that CIRT has a promising curative effect on certain types of breast cancer with limited adverse effects. However, only approximately 34 clinical practices have been reported by 2022. There is a huge gap between the large population of breast cancer and the clinical research progress (Figure). Although the time to complete the response was longer than expected in some studies, the persistent responses and satisfactory safety profile provided the rationale for further research on this new therapy. Large randomized phase III trials should be conducted in the future to support the efficacy and safety of CIRT for breast cancer.

**Figure:**
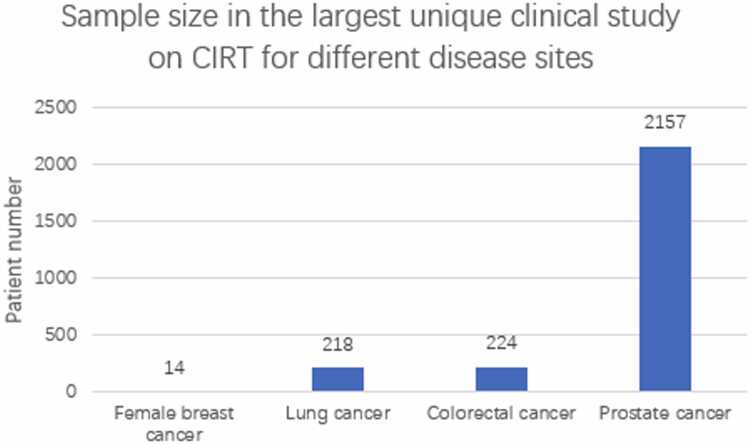
Cancer incidence and patients treated with CIRT. There were some key barriers to performing studies of CIRT on breast cancer, including anatomical challenges, reliance on established treatments, high cost, low instrument availability, and underestimated benefit of CIRT on breast cancer. Abbreviation: CIRT, carbon-ion radiation therapy.

Most clinical studies are focused on early-stage breast cancer, and patients with advanced tumors respond to CIRT well,^24^ indicating that more studies on selected tumor types are needed in the future.

However, based on the largest number of patients involved in recent clinical studies, current CIRT development in the treatment of breast cancer is slower than the other 3 top popular cancer types (Figure).^24^, ^25^, ^26^, ^27^

Firstly, the anatomical location of breast tumors presents unique challenges for CIRT. Breast tissue is close to critical organs such as the heart and lungs, making it difficult to deliver high doses of radiation without causing significant collateral damage. The precision required to avoid these organs while targeting the tumor requires advanced techniques and technologies, which are still under development and not widely available. Carbon ions have a high LET, which means they deposit most of their energy at the end of their range, known as the Bragg peak. Beyond the Bragg peak, there is a fragmentation tail where some energy is still deposited. The fragmentation tail can potentially affect healthy tissue beyond the intended target area, although the risk is lower than with traditional RT. However, CIRT is still promising since the Bragg Peak phenomenon allows precise dose delivery to the tumor while minimizing damage to healthy tissues, and the clinical outcomes of CIRT for hepatocellular carcinoma have shown excellent local control rates with minimal side effects.^10^ Of note, the movement of the tumor due to breathing and other motions can affect the accuracy of the treatment. To mitigate this risk, immobilization devices and respiratory gating systems may be used to synchronize the treatment with the patient's breathing cycle. Recent developments in artificial intelligence also demonstrated the feasibility of patient-specific treatment planning for heavy ion RT by using a deep learning approach.^28^

Secondly, there is a historical reliance on conventional treatment modalities such as surgery, chemotherapy, and conventional RT, which have been extensively studied and are well-established in the treatment of breast cancer.^29^ The established protocols and long-term data supporting these treatments make it challenging for new modalities like CIRT to gain immediate acceptance. However, patients with locally advanced or recurrent breast cancer may benefit significantly from CIRT due to its high precision and potential for better local control compared to conventional RT. Triple-negative breast cancer patients, who typically have limited treatment options and poorer prognosis, may also benefit from the enhanced biological effectiveness of CIRT.^30^ The focus of future clinical research also includes how CIRT can be combined with other systemic treatments, chemotherapy, and targeted therapy to improve clinical efficacy. Combining CIRT with immunotherapy or targeted therapies could enhance treatment efficacy. For instance, combining CIRT with immune checkpoint inhibitors may boost the immune response against cancer cells, providing a synergistic effect.^12^ Immunotherapy combined with photon RT has been successfully used in the clinic, and the effect and mechanism of immunotherapy combined with particle RT are gaining more interest.^31^ The combination of CIRT with Hh inhibitor, dual tyrosine kinase inhibitor, and PARP inhibitor for breast cancer is promising, as demonstrated in the in vitro studies. However, clinical evidence is still needed.

Thirdly, the cost of CIRT is still high, and there are a limited number of facilities, which may be a barrier to large clinical trials. Carbon-ion RT requires specialized equipment and facilities, which are not as prevalent as those for conventional RT. The investment required to establish and maintain these facilities is substantial, limiting their accessibility. As of early 2023, a total of 14 institutions are regularly performing CIRT, with half of them located in Japan. Four facilities are under construction, and 2 facilities are in the planning stage.^31^ Given the slow machine construction and high treatment cost, research on device miniaturization and price reduction is needed in the future. Facilities about one third of the size and less than half the cost of HIMAC facilities have been made and put into operation. Furthermore, funding support from government and private sectors, as well as international collaborations to share resources and expertise, may help boost the development of CIRT.

Finally, the benefit of CIRT on breast cancer is underestimated due to the limited number of studies. Researchers prioritized the studies on more traditionally radio-resistant tumors, such as sarcomas and head and neck cancers, where CIRT has shown significant benefits. In this review, we tried to summarize the promising benefit of CIRT for breast cancer, hoping to raise awareness and encourage its adoption.

## Conclusions

Although nonclinical and clinical studies demonstrated promising treatment effects with acceptable and manageable risks, the physical and biological characteristics of CIRT in treating breast cancer have not been thoroughly studied. Research on CIRT for breast cancer has been limited due to the high cost and limited number of institutions performing CIRT. The combination of CIRT with modern breast cancer therapies is promising in nonclinical studies, but clinical evidence is still needed. Furthermore, future endeavors ought to include conducting randomized phase III trials to substantiate the efficacy and safety of CIRT in the treatment of breast cancer.

## Funding

This research was supported by the 10.13039/501100001809National Natural Science Foundation of China to Xiaohua Pei (grant number 81774319).

## Author Contributions

All authors were involved in the process of collecting and reading the papers and unanimously agreed to the manuscript.

## Declaration of Conflicts of Interest

The authors declare the following financial interests/personal relationships which may be considered as potential competing interests: Xiaohua Pei reports financial support was provided by National Natural Science Foundation of China. If there are other authors, they declare that they have no known competing financial interests or personal relationships that could have appeared to influence the work reported in this paper.
